# Retraction: Comparative Effectiveness of Endoscopic Versus Pharmacological Interventions for Variceal Rebleeding in Cirrhosis: A Systematic Review

**DOI:** 10.7759/cureus.r187

**Published:** 2025-07-16

**Authors:** Muath M Dabas, Muhammad Maqbool, Adees W Bedros, Hiba Mazhar, Papuna Papuashvili, Muhammad Umar, Aqsa B Bajwa, Dhruvi H Patel, Nada B Abushalha, Abid Khattak, Junaid Ahmed, Asma Mehdi

**Affiliations:** 1 Surgery, The University of Jordan, Amman, JOR; 2 Internal Medicine, Shaheed Mohtarma Benazir Bhutto Medical College Lyari, Karachi, PAK; 3 Internal Medicine, The University of Jordan, Amman, JOR; 4 Gastroenterology, Pakistan Kidney and Liver Institute, Lahore, PAK; 5 Internal Medicine, Tbilisi State Medical University, Tbilisi, GEO; 6 Internal Medicine, Faisalabad Medical University, Faisalabad, PAK; 7 Internal Medicine, Shalamar Medical and Dental College, Riyadh, SAU; 8 Internal Medicine, Civil Hospital, Surat, IND; 9 Acute Medicine, Sherwood Forest Hospitals NHS Foundation Trust, Sutton-in-Ashfield, GBR; 10 Internal Medicine, Chandka Medical College Hospital, Larkana, PAK; 11 Surgery, Rawalpindi Medical University, Rawalpindi, PAK

The Editors-in-Chief have retracted this article following an ethics complaint and internal investigation. This article was linked to an authorship-for-sale scheme, with evidence showing the article was advertised in a group offering paid authorship. Several listed authors admitted to having no role in the research or writing, confirming the use of unethical authorship practices. As a result, the credibility of the article is compromised and retraction is required.

